# Effect of Ishophloroglucin A Isolated from *Ishige okamurae* on In Vitro Osteoclastogenesis and Osteoblastogenesis

**DOI:** 10.3390/md21070377

**Published:** 2023-06-26

**Authors:** Su-Hyeon Cho, Hyun-Soo Kim, Hye-Yeon Jung, Jae-Il Park, You-Jee Jang, Juhee Ahn, Kil-Nam Kim

**Affiliations:** 1 Chuncheon Center, Korea Basic Science Institute (KBSI), Chuncheon 24341, Republic of Korea; chosh93@kbsi.re.kr; 2 Department of Medical Biomaterials Engineering, College of Biomedical Sciences, Kangwon National University, Chuncheon 24341, Republic of Korea; 3 National Marine Biodiversity Institute of Korea, Seocheon 33662, Republic of Korea; gustn783@mabik.re.kr; 4 Gwangju Center, Korea Basic Science Institute (KBSI), Gwangju 61751, Republic of Korea; jhy0724@kbsi.re.kr (H.-Y.J.); jaeil74@kbsi.re.kr (J.-I.P.); 5 Department of Biomedical Laboratory Science, Honam University, Gwangju 62399, Republic of Korea; kshowmin80@honam.ac.kr; 6 Department of Bio-Analysis Science, University of Science & Technology, Daejeon 34113, Republic of Korea

**Keywords:** Ishophlorogulcin A, *Ishige okamurae*, osteoclastogenesis, osteoblastogenesis, RAW 264.7 cells, MG-63 cells

## Abstract

The balance between bone-resorbing osteoclasts and bone-forming osteoblasts is essential for the bone remodeling process. This study aimed to investigate the effect of Ishophloroglucin A (IPA) isolated from *Ishige okamurae* on the function of osteoclasts and osteoblasts in vitro. First, we demonstrated the effect of IPA on osteoclastogenesis in receptor activator of nuclear factor κB ligand (RANKL)-induced RAW 264.7 cells. IPA inhibited the tartrate-resistant acid phosphatase (TRAP) activity and osteoclast differentiation in RANKL-induced RAW 264.7 cells. Moreover, it inhibited the RANKL-induced osteoclast-related factors, such as TRAP, matrix metalloproteinase-9 (MMP-9), and calcitonin receptor (CTR), and transcription factors, such as nuclear factor of activated T cells 1 (NFATc1) and c-Fos. IPA significantly suppressed RANKL-activated extracellular signal-regulated kinase (ERK), and NF-κB in RAW 264.7 cells. Our data indicated that the ERK and NF-κB pathways were associated with the osteoclastogenesis inhibitory activity of IPA. Next, we demonstrated the effect of IPA on osteoblastogenesis in MG-63 cells. IPA significantly promoted alkaline phosphatase (ALP) activity in MG-63 cells, along with the osteoblast differentiation-related markers bone morphogenetic protein 2 (BMP2), type 1 collage (COL1), p-Smad1/5/8, and Runx2, by activating the MAPK signaling pathways. Taken together, the study indicated that IPA could be effective in treating bone diseases, such as osteoporosis.

## 1. Introduction

Bone remodeling is an important process that maintains the normal skeletal structure and function of the body [1]. Osteoclasts and osteoblasts are the two main cells involved in bone remodeling [2]. While osteoclasts remove aged or damaged bones, osteoblasts form new bones [1]. However, an imbalance between osteoclasts and osteoblasts promotes the development of destructive bone diseases, such as osteoporosis [2]. Generally, estrogen, calcitonin, and bisphosphonates are used as anti-resorptive agents to prevent bone loss [3]. However, estrogen treatment continues for more than five years and can cause severe side effects, such as increased body weight, endometrial cancer, breast cancer, and irregular uterine bleeding [3,4]. Bisphosphonates, bone-resorbing drugs, such as alendronate, risedronate, ibandronate, and zoledronate, are used to treat osteoporosis, and also have side effects, such as bisphosphonate-related osteonecrosis of the jaw and atypical femoral fractures [4]. Parathyroid hormone (PTH) plays a role in bone formation, and teriparatide has been used as an anabolic agent to increase bone mass [5,6]. Romosozumab is the only drug that acts on both bone resorption and formation [7]. Therefore, the development of natural products for functional foods and drugs would be crucial for the treatment of osteoporosis without any side effects.

Osteoclasts and osteoblasts involved in bone remodeling are activated by different mechanisms. Osteoclasts are multinucleated cells that are formed by the fusion of monocytes with macrophage-derived precursors; they are responsible for bone resorption [8]. The maturation and differentiation of osteoclasts from monocyte/macrophage-derived precursors are associated with the receptor activator of nuclear factor-κB (RANK) ligand (RANKL)/RANK/osteoprotegerin (OPG) signaling pathway [9]. In this pathway, RANKL binds to RANK and activates the downstream signaling pathways, such as the mitogen-activated protein kinase (MAPK) and nuclear factor kappa-B (NF-κB) pathways [9]. Subsequently, phosphorylation of MAPK and NF-κB activates nuclear factor of activated T cells 1 (NFATc1) and activator protein 1. Consequently, the cell expresses osteoclast-related factors, including tartrate-resistant acid phosphatase (TRAP), matrix metalloproteinase-9 (MMP-9), calcitonin receptor (CTR), and cathepsin K [10]. 

Osteoblasts are derived from mesenchymal stem cells (MSCs) and play a crucial role in bone formation [11]. Bone formation by osteoblasts involves various mechanisms, including bone morphogenetic protein (BMP)/Smad and MAPK signaling pathways [12]. The mechanisms activate Runt-related transcription factor 2 (Runx2) as the main transcription factor [13]. Runx2 induces the expression of osteoblast-related proteins, such as type 1 collagen (COL1) and alkaline phosphatase (ALP) [13]. 

Brown algae contains diverse biological compounds, including polysaccharides, mannitol, unsaturated fatty acids, phlorotannins, fucoxanthin, gibberellin, and vitamins [14]. Phlorotannins in brown algae exhibit a variety of biological activities, including anti-inflammatory, antioxidant, and anti-diabetic activities [15,16,17]. Ishophloroglucin A (IPA) is a major phlorotannin compound of *Ishige okamurae*, together with diphlorethohydroxycarmalol (DPHC). Previous studies have reported that phlorotannins effectively inhibit osteoporosis. Diekcol inhibits RANKL-induced osteoclastogenesis through suppressing the activation of extracellular signal-regulated kinase (ERK), JNK, and NF-κB [18]. Triphlorethol-A, eckol, and dieckol ameliorated adipogenic differentiation and enhanced osteoblast differentiation in vitro [19]. In addition, DPHC inhibits osteoclastogenesis by downregulating the NF-κB signaling [20]. Studies have reported that IPA is involved in various biological activities, including anti-α-glucosidase and anti-melanogenesis, and inhibits obesity and peripheral fat accumulation [21,22]. However, the osteoclastogenesis-inhibitory activity of IPA and the mechanisms underlying osteoporosis remain to be investigated. In this study, we evaluated the in vitro inhibitory effect of IPA on osteoporosis and elucidated the mechanisms underlying the effect.

## 2. Results

### 2.1. Effect of IPA on Cell Viability and TRAP Activity in RANKL-Induced RAW 264.7 Cells

**Figure 1:**
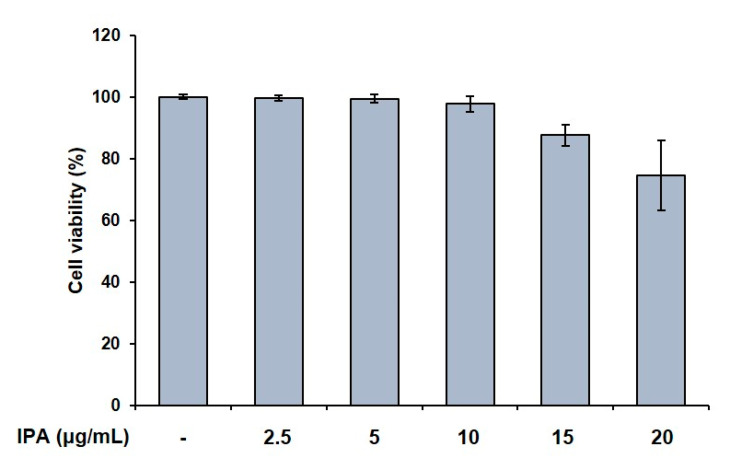
Effect of IPA on cell viability in RAW 264.7 cells. Cell viability was assessed by MTT assay. All results were expressed as the mean ± standard deviation (SD; n = 3) of triplicate experiments. Statistical analysis was calculated using one-way analysis of variance (ANOVA).

**Figure 2:**
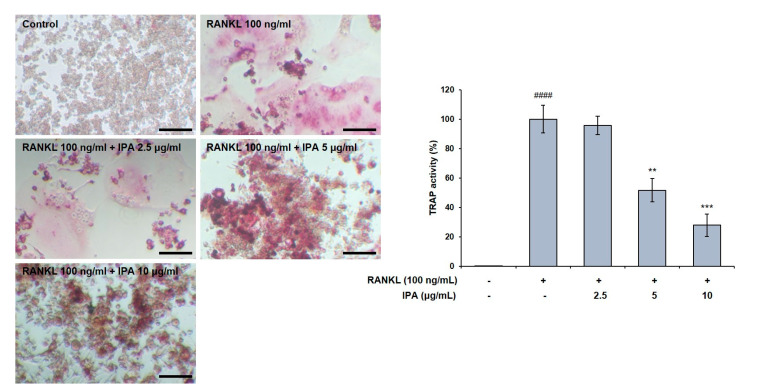
Effect of IPA on TRAP activity in RANKL-induced RAW 264.7 cells. TRAP activity was examined using TRAP staining (scale bar: 100 μm). All results were expressed as the mean ± SD (n = 3) of triplicate experiments; #### *p* < 0.0001 compared with the control group (without RANKL and IPA treatment). ** *p* < 0.01, *** *p* < 0.001, and compared with the RANKL-stimulated group. Statistical analysis was calculated using one-way ANOVA.

We first confirmed the effect of IPA on cell viability in RAW 264.7 cells, and found it to not be cytotoxic up to 10 μg/mL (Figure 1). We used these non-cytotoxic concentrations for the subsequent experiments. We next demonstrated the effect of these compounds on the TRAP activity in the RAW 264.7 cells, and observed that the RANKL treatment induced osteoclast differentiation. Moreover, the RANKL-treated cells exhibited higher TRAP activity than the control cells. Amounts of 5 and 10 μg/mL of IPA significantly inhibited the RANKL-induced TRAP activity (5 μg/mL, *p* < 0.01; 10 μg/mL, *p* < 0.001, Figure 2).

### 2.2. IPA Inhibits RANKL-Induced Expression of Osteoclast Differentiation-Related Protein in RAW 264.7 Cells

**Figure 3:**
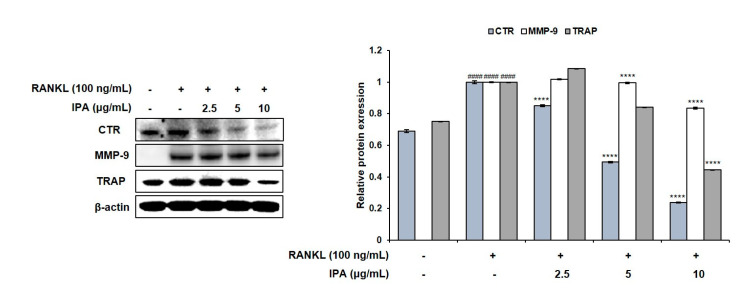
Effect of IPA on expression of osteoclast-related factors in RANKL-induced RAW 264.7 cells. Protein expression was assessed using Western blot analysis. All results were expressed as the mean ± SD (n = 3) of triplicate experiments; #### *p* < 0.0001 compared with the control group (without RANKL and IPA treatment). **** *p* < 0.0001 compared with the RANKL-stimulated group. Statistical analysis was calculated using one-way ANOVA.

We investigated the effect of IPA and RANKL on the expression levels of the factors involved in osteoclast differentiation in the RAW 264.7 cells. Although RANKL induced the expression of CTR, MMP-9, and TRAP in the IPA-treated cells compared to those in the untreated cells, IPA significantly downregulated the expression levels in a concentration-dependent manner (*p* < 0.0001, Figure 3).

### 2.3. IPA Inhibits RANKL-Induced Expression of Osteoclast-Differentiation-Related Transcriptional Factors in RAW 264.7 Cells

**Figure 4:**
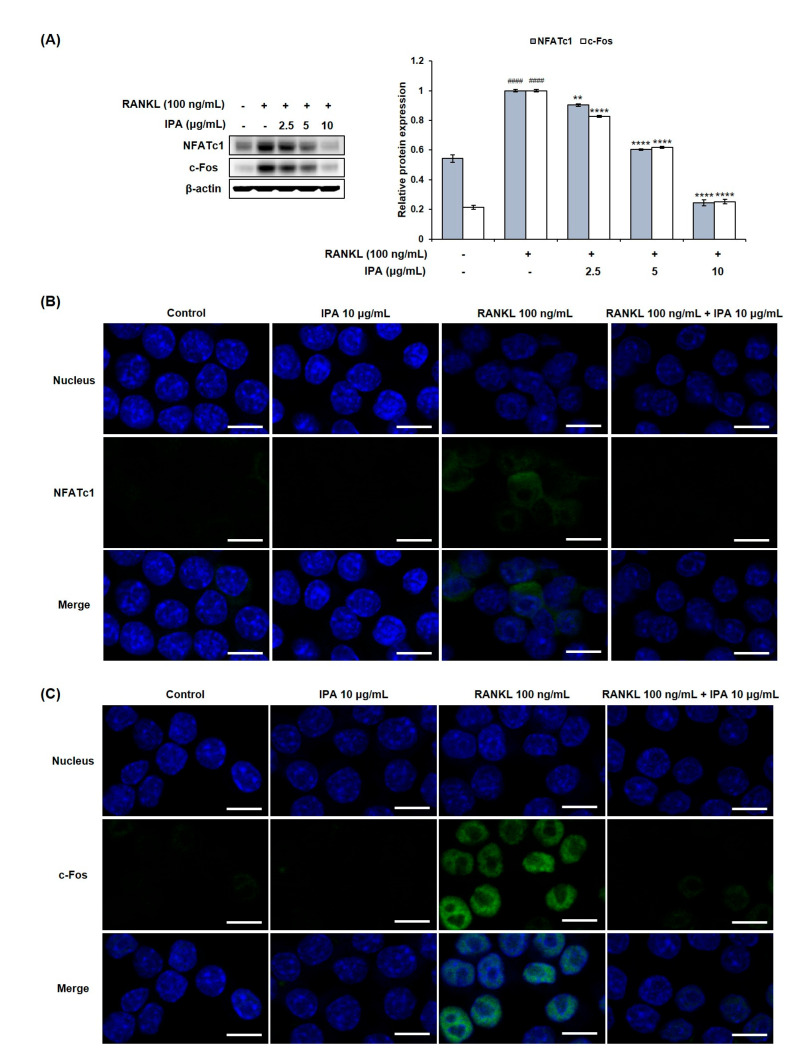
Effect of IPA on expression of osteoclast-related transcription factors in RANKL-induced RAW 264.7 cells. (**A**) Protein expression was assessed using Western blot analysis. (**B**) The expression of NFATc1 and c-Fos was observed using IF staining. These data were observed with an anti-NFATc1 and Alexa Fluor 488 goat anti-mouse antibody by LSM700 Zeiss confocal laser scanning microscope (scale bar: 20 μm). (**C**) All results were expressed as the mean ± SD (*n* = 3) of triplicate experiments; #### *p* < 0.0001 compared with the control group (without RANKL and IPA treatment). ** *p* < 0.01 and **** *p* < 0.0001 compared with the RANKL-stimulated group. Statistical analysis was calculated using one-way ANOVA.

We investigated the effect of IPA on the expression levels of osteoclastogenesis-related key transcription factors in the RANKL-induced RAW 264.7 cells by Western blotting and immunofluorescence (IF) staining. RANKL induced the expression of NFATc1 and c-Fos in the RANKL-treated cells compared to that in the untreated cells (Figure 4). However, IPA significantly down-regulated the NFATc1 and c-Fos expression in a concentration-dependent manner.

### 2.4. IPA Inhibits RANKL-Induced Activation of NF-κB and ERK in RAW 264.7 Cells

**Figure 5:**
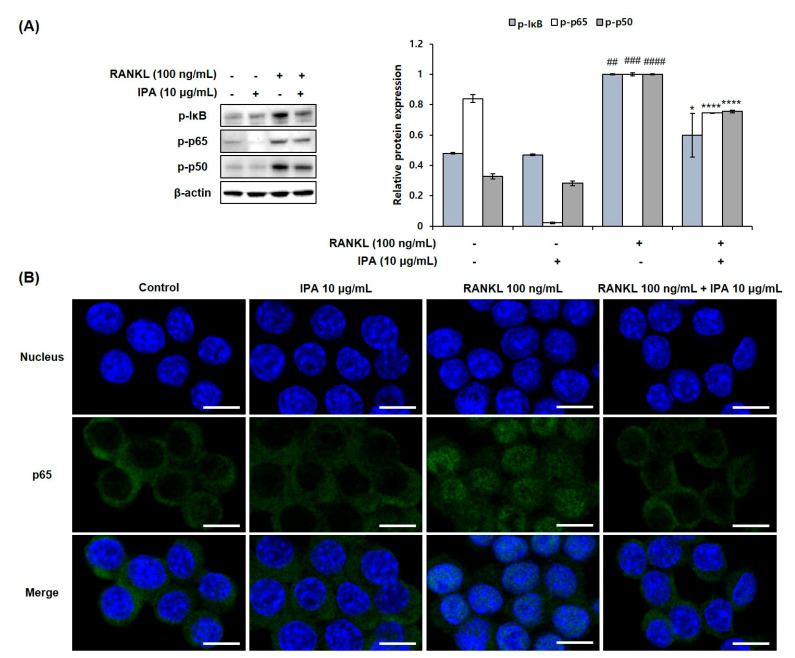
Effect of IPA on activation of NF-κB in RANK-induced RAW 264.7 cells. (**A**) Protein expression was assessed using Western blot analysis. (**B**) The activation of p65 was observed using IF staining. These data were observed with an anti-NFATc1 and Alexa Fluor 488 goat anti-mouse antibody by LSM700 Zeiss confocal laser scanning microscope (scale bar: 20 μm). All results were expressed as the mean ± SD (n = 3) of triplicate experiments; ## *p* < 0.01, ### *p* < 0.001, and #### *p* < 0.0001 compared with the control group (without RANKL and IPA treatment). * *p* < 0.05 and **** *p* < 0.0001 compared with the RANKL-stimulated group. Statistical analysis was calculated using one-way ANOVA.

**Figure 6:**
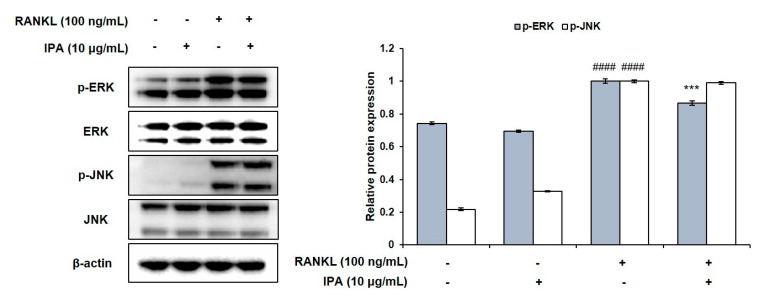
Effect of IPA on activation of MAPK in RANKL-induced RAW 264.7 cells. Protein expression was assessed using Western blot analysis. All results were expressed as the mean ± SD (n = 3) of triplicate experiments; #### *p* < 0.0001 compared with the control group (without RANKL and IPA treatment). *** *p* < 0.001 compared with the RANKL-stimulated group. Statistical analysis was calculated using one-way ANOVA.

We determined the inhibitory effect of IPA on NF-κB and MAPK in the RANKL-stimulated RAW 264.7 cells. RANKL induced the phosphorylation of extracellular signal-regulated kinase (ERK), IκB, p50, and p65 (Figure 5A). However, 10 μg/mL IPA significantly inhibited the RANKL-induced activation of ERK (Figure 6) and NF-κB (Figure 5A).

### 2.5. IPA Promotes Osteoblasts Differentiation in MG-63 Cells

**Figure 7:**
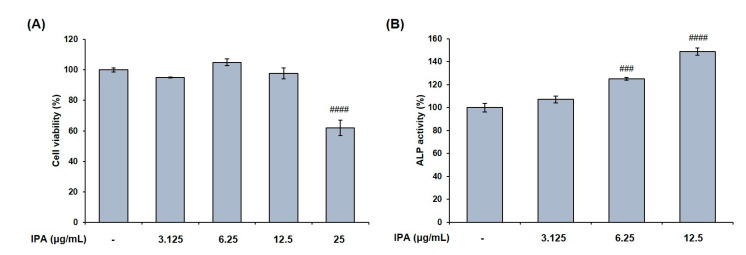
Effect of IPA on (**A**) cell viability and (**B**) ALP activity in MG-63 cells. Cell viability was assessed by MTT assay. ALP activity was measured using substrate, *p*-nitrophenyl phosphate (*p*-NPP). All results were expressed as the mean ± SD (n = 3) of triplicate experiments; ### *p* < 0.001 and #### *p* < 0.0001 compared with the control group.; Statistical analysis was calculated using one-way ANOVA.

**Figure 8:**
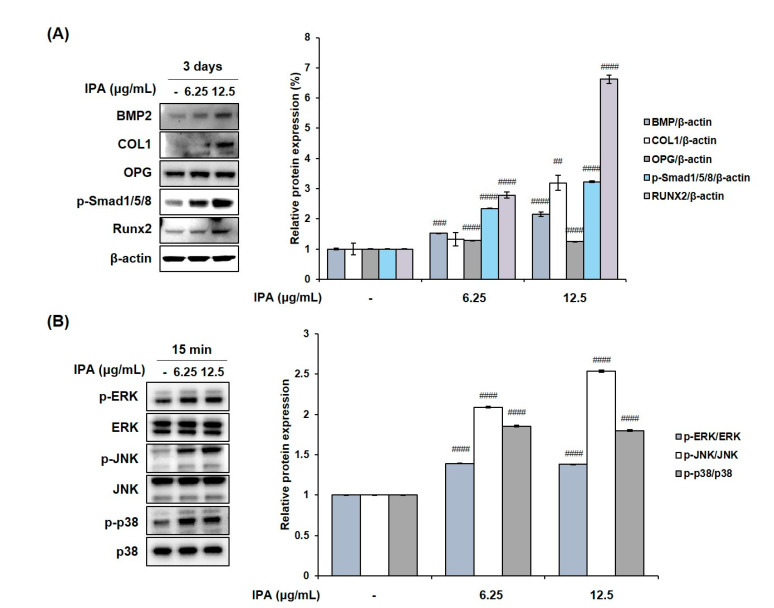
Effect of IPA on (**A**) expression of osteoblast-related factors and (**B**) activation of MAPK in MG-63 cells. Protein expression was assessed using Western blot analysis. All results were expressed as the mean ± SD (n = 3) of triplicate experiments; ## *p* < 0.01, ### *p* < 0.001, and #### *p* < 0.0001 compared with the control group.; Statistical analysis was calculated using one-way ANOVA.

We measured the cell viability after treating MG-63 cells with various concentrations of IPA. The latter was not cytotoxic at concentrations up to 12.5 μg/mL (Figure 7A). These concentrations were used to prove the effect of IPA on osteoblast differentiation. Next, we confirmed the activity of osteoblast marker ALP, and determined the degree of mineralization after IPA treatment for 3 d. Results revealed that IPA significantly increased the ALP activity at concentrations ranging from 3.125 to 12.5 μg/mL compared to that in the control group (Figure 7B). Next, we examined the mechanisms of osteoblast differentiation using Western blot analysis. Osteoblast differentiation markers, BMP2, COL1, OPG, p-Smad1/5/8, and Runx2 levels significantly increased in the cells treated with IPA compared to that in the control group (Figure 8A). In addition, IPA phosphorylated ERK, JNK, and p38 in the cells (Figure 8B).

## 3. Discussion

The balance between bone resorptive osteoclasts and bone-forming osteoblasts is essential for bone remodeling [1]. Previous studies had reported that phlorotannins have anti-osteoporotic activity. For instance, Ihn et al. (2017) demonstrated that DPHC affects RANKL-induced osteoclast differentiation by blocking the NF-κB signaling pathway in vitro [22]. Additionally, Karadeniz et al. (2015) established that triphlorethol-A, eckol, and dieckol have potent anti-adipogenesis and osteoblastogenesis effects in cells [19]. Therefore, we examined the effect of phlorotannin IPA on the osteoclast differentiation in vitro to confirm its effectiveness.

RANKL protein is a member of the tumor necrosis factor receptor family and regulates osteoclast differentiation and function [23]. In this study, we induced the differentiation of RAW 264.7 cells into osteoclasts via RANKL treatment in accordance with a previously published study [24]. Subsequently, we determined the in vitro effect of IPA on TRAP activity and osteoclast differentiation. We observed that IPA (10 μg/mL) significantly inhibited the TRAP activity and osteoclast differentiation.

Osteoclast differentiation and bone resorption occur excessively in multiple bone disorders, such as periodontitis, rheumatoid arthritis, and osteoporosis [25]. The binding of RANKL to its receptor RANK recruits TRAF6 and activates the downstream signaling pathways. Particularly, NF-κB and MAPK signaling pathways play crucial roles in osteoclastogenesis [26]. The recruited TNF receptor associated factor 6 (TRAF6) activates the inhibitor of κB (IκB) kinase (IKK) and the MAPK cascades [26]. Subsequently, IKK activation induces the phosphorylation and degradation of IκB, in turn activating the p65/p50 heterodimer [27]. Recruitment of TRAF6 also activates MAPK. Notably, ERK, which is a component of the MAPK pathway, plays an important role in osteoclast differentiation and function [25]. Activated NF-κB and ERK activate c-Fos and induce the expression of NFATc1, a member of the NFAT family [19]. Consequently, the expression levels of osteoclast-related factors, such as CTR, TRAP, and MMP-9, are up-regulated [10]. We identified the factors related to osteoclast differentiation by examining the protein expression levels. In this regard, IPA significantly suppressed the RANKL-induced expression levels of CTR, TRAP, and MMP-9. Moreover, it inhibited the expression of NFATc1 and c-Fos. We also investigated the mechanisms underlying the inhibitory effects of IPA on osteoclastogenesis. While RANKL induced the phosphorylation of NF-κB and ERK, IPA significantly suppressed them. Thus, our study demonstrated that IPA is an effective inhibitor of osteoclastogenesis because it suppressed the expression of NFATc1 and c-Fos, and the activation of NF-κB and ERK.

Osteoblasts, derived from bone marrow mesenchymal stem cells (MSCs), play an important role in bone formation and remodeling [28]. Human osteoblast-like MG-63 cells have previously been used to study osteoblast differentiation [28]. Hence, we confirmed the effect of IPA on the ALP activity in MG-63 cells. ALP, as a byproduct of osteoblast activity, is a phenotype marker for the early differentiation of osteoblasts [29]. In the present study, IPA significantly increased the ALP activity compared to that in the control group. The results collectively suggested that IPA induces osteoblast differentiation and bone formation.

To elucidate the mechanisms of osteoblast differentiation, we examined osteoblast-related protein expression using Western blot analysis. BMP2 plays a crucial role as an inducer of differentiation in pre-osteoblasts differentiation via the canonical BMP/Smad and non-canonical BMP pathways [30]. Binding of BMP2 to type II BMP receptors initiates the activation of receptor-regulated SMAD (R-SMAD) proteins (Smad1/5/8) [13]. Activated Smad1/5/8 forms a complex with Smad4, which is translocated into the nucleus to express osteoblast differentiation-related genes [30]. In addition to the BMP/Smad signaling pathway, the MAPK signaling pathway functions as a non-canonical BMP2 pathway [30]. MAPKs are composed of ERK, JNK, and p38, and regulate various cellular functions, including cell death, proliferation, migration, motility, survival, and terminal differentiation [30]. BMP2 promotes the activation of MAPK, including ERK, JNK, and p38 [30]. Runx2, ALP, COL1, and OPG regulate osteoblast differentiation and bone formation [10,13,31,32,33]. Our study demonstrated that IPA activated the expression of osteoblast differentiation-related factors, including BMP2, COL1, OPG, p-Smad1/5/8, and Runx2, via the canonical BMP2/Smad and non-canonical BMP2/MAPK signaling pathways.

In conclusion, this study demonstrated the effect of IPA on osteoporosis in vitro. Remarkably, IPA significantly inhibited RANKL-stimulated osteoclastogenesis via suppression of NFATc1 and c-Fos by blocking the activation of the NF-κB and ERK signaling pathways. Moreover, it promoted osteoblast differentiation and mineralization by activating Runx2 via the promotion of the Wnt/β-catenin, BMP-2/Smad, and BMP-2/MAPK signaling pathways. The results collectively showed that IPA could potentially be used in drug development or in functional foods to treat osteoporosis by regulating the bone remodeling process.

## 4. Materials and Methods

### 4.1. Sample Preparation

**Figure 9:**
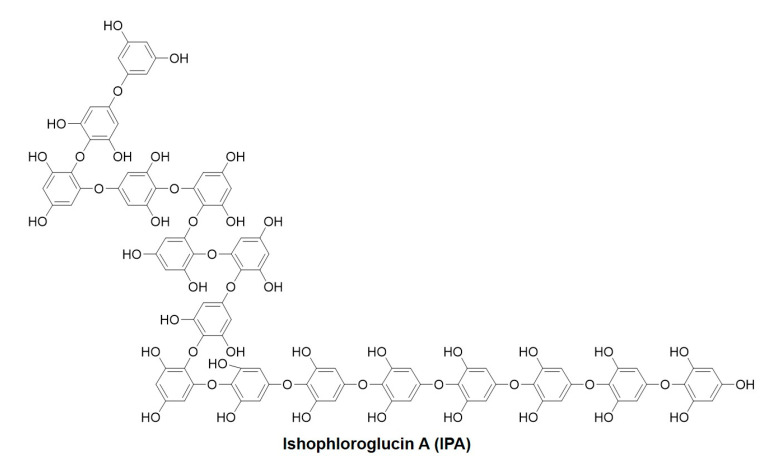
Chemical structure of Ishophloroglucin A (IPA).

*I. okamurae* samples were collected from Jeju Island and washed thrice with tap water to remove salt and sand from the sample surface. The washed samples were then freeze-dried. The samples were then homogenized using a grinder. Powered *I. okamurae* (100 g) was subjected to extraction using 70% ethanol (1 L) with continuous agitation at 37 °C, and the solvent was removed under vacuum using a rotary evaporator. The extract was partitioned with ethyl acetate to obtain the ethyl acetate fraction. The ethyl acetate fraction was then subjected to high-performance centrifugal partition chromatography (HPCPC) as previously described [34]. Approximately 20 mg was obtained with >80% purity using HPCPC. Figure 9 shows the chemical structure of IPA.

### 4.2. Cell Culture

We purchased the murine macrophage cell line RAW 264.7 from the Korean Cell Line Bank (KCLB, Seoul, Republic of Korea), and human osteoblast-like cell line MG-63 from American Type Culture Collection (ATCC, Manassas, VA, USA). We cultured the cells in Dulbecco’s Modified Eagle’s Medium (DMEM; Welgene, Daegu, Republic of Korea) supplemented with 10% fetal bovine serum (FBS; Welgene, Daegu, Republic of Korea) and 1% of 100 X antibiotic-antimycotic (10,000 units/mL penicillin, 10,000 μg/mL streptomycin, and 25 μg/mL amphotericin B; Gibco BRL, CA, USA) at 37 °C in a humidified incubator under 5% CO_2_.

### 4.3. MTT Assay

We seeded 2 × 10^4^ RAW 264.7 cells/mL in a 96-well plate. After 24 h, the cells were incubated with various concentration of IPA (2.5, 5, 10, 15, and 20 μg/mL) for 5 days. MG-63 cells were seeded in a 96-well plate at a concentration of 1.5 × 10^4^ cells/well, and treated with various concentration of IPA (3.125, 6.25, 12.5, and 25 μg/mL) for 3 days. MTT assay were performed according to the method of Cho et al. [35]. Absorbance of the formazan crystals was measured at 540 nm using a SpectraMax M2/M2e spectrophotometer (Molecular Devices, Sunnyvale, CA, USA).

### 4.4. Tartrate-Resistant Acid Phosphatase (TRAP) Staining

We seeded RAW 264.7 cells at 2 × 10^4^ cells/mL in a 24-well plate and incubated them for 24 h. The cells were pre-treated with IPA (2.5, 5, and 10 μg/mL) for 2 h. Subsequently, the cells were treated with 100 ng/mL RANKL (Sigma-Aldrich, St. Louis, MO, USA) in the presence of IPA for 5 days. The spent medium was replaced with fresh medium every 2 days. We measured TRAP activity using a TRAP staining kit (Cosmo Bio Co., Ltd., Tokyo Japan) according to the manufacturer’s protocol. TRAP-positive cells were visualized and counted under a light microscope (Carl Zeiss, Jena, Germany).

### 4.5. Alkaline Phosphatase (ALP) Activity

MG-63 cells were seeded in a 96-well plate at a concentration of 1.5 × 10^4^ cells/well and incubated for 24 h. The cells were treated with IPA (3.125, 6.25, and 12.5 μg/mL) for 3 days. ALP activity was then measured according to the method described by Baek et al. [36].

### 4.6. Western Blot Analysis

We performed Western blotting by incubating 2 × 10^4^ RAW 264.7 cells/mL for 24 h in a 6-well plate. Following this, we pre-treated the RAW 264.7 cells with various concentrations of IPA for 2 h and subsequently treated them with 100 ng/mL RANKL in the presence of IPA for the indicated times (osteoclast-related factors: 5 days; transcription factors: 9 h, NF-κB: 5 min; MAPK: 20 min). Every 2 days, we replaced the spent medium with fresh medium. MG-63 cells were treated with various concentration of IPA (6.25 and 12.5 μg/mL) for the indicated times (osteoblast-related factors: 3 days; MAPK: 15 min). Protein expression was analyzed as described by Cho et al. with some modification [35]. The membranes were incubated overnight at 4 °C with the following primary antibodies: anti-TRAP (Abcam, Waltham, MA, USA), anti-MMP-9 (Abcam, Waltham, MA, USA), anti-CTR (Abcam, Waltham, MA, USA), anti-NFATc1 (BD Pharmingen^TM^, San Diego, CA, USA), anti-c-Fos (Cell Signaling Technology, Beverly, MA, USA), anti-c-Jun (Cell Signaling Technology, Beverly, MA, USA), anti-phospho-ERK (Cell Signaling Technology, Beverly, MA, USA), anti-ERK (Cell Signaling Technology, Beverly, MA, USA), anti-phospho-JNK (Cell Signaling Technology, Beverly, MA, USA), anti-phospho-IκB (Cell Signaling Technology, Beverly, MA, USA), anti-phospho-p65 (Cell Signaling Technology, Beverly, MA, USA), anti-phospho-p50 (Santa Cruz Biotechnology, CA, USA), anti-BMP2 (Abcam, Waltham, MA, USA), anti-COL1A1 (Santa Cruz Biotechnology, CA, USA), anti-OPG (Abcam, Waltham, MA, USA), anti-phospho Smad1/Smad5/Smad8 (Sigma Aldrich, St. Louis, MO, USA), and anti-β-actin (Santa Cruz Biotechnology, CA, USA). Subsequently, the membranes were incubated for 2 h with the following secondary antibodies at room temperature: anti-rabbit IgG, horseradish peroxidase (HRP)-linked antibody, anti-mouse IgG, and HRP-linked antibody (Cell Signaling Technology, Beverly, MA, USA). Membranes were stripped to remove primary and secondary antibodies and re-probed using a stripping buffer (Dyne Bio, Seongnam, Republic of Korea).

### 4.7. Confocal Laser Scanning Microscopy (CLSM)

We seeded 5 × 10^4^ RAW 264.7 cells/mL in a confocal chamber slide and pre-treated them with 10 μg/mL IPA for 2 h. Subsequently, we treated the cells with 100 ng/mL RANKL in the presence of IPA for 9 h and 5 min. IF staining was performed as described by Cho et al. with some modification [35]. The cells were incubated overnight at 4 °C with the following primary antibodies: anti-NFATc1 (1:100), anti-c-Fos (1:100), and anti-p65 (1:100). Subsequently, the cells were incubated for 1.5 h with the following secondary antibodies at room temperature: Alexa fluor488-labeled goat anti-rabbit IgG (H + L) cross-adsorbed secondary antibody and Alexa fluor488-labeled goat anti-mouse IgG (H + L) cross-adsorbed secondary antibody (1:800; Thermo Fisher Scientific, Inc., Foster City, CA, USA). Nuclei were stained with 40 μg/mL Hoechst 33342 (Sigma-Aldrich, St. Louis, MO, USA) for 10 min at room temperature. Eventually, fluorescent signals were detected using an LSM 700 Zeiss confocal laser scanning microscope (Carl Zeiss, Jena, Germany).

### 4.8. Statistical Analysis

The data were expressed as mean ± standard deviation (SD) and were analyzed by one-way ANOVA with Tukey’s post hoc test. Data were considered statistically significant at *p* < 0.05. All statistical tests were performed using GraphPad Prism software (version 8.0; GraphPad Software, San Diego, CA, USA).

## Data Availability

The data presented in this study are available on request from the corresponding author.
